# The Effects of Exergames on Physical Fitness, Body Composition and Enjoyment in Children: A Six-Month Intervention Study

**DOI:** 10.3390/children11101172

**Published:** 2024-09-26

**Authors:** Santo Marsigliante, Giulia My, Gianmarco Mazzotta, Antonella Muscella

**Affiliations:** Department of Biological and Environmental Science and Technologies [DiSTeBA], University of Salento, 73100 Lecce, Italy; santo.marsigliante@unisalento.it (S.M.);

**Keywords:** exergaming intervention, physical education, physical fitness, psychological effects

## Abstract

Background/Objectives: Physical inactivity in children can lead to decreased physical fitness and reduced enjoyment of physical activity. This study aimed to evaluate the impact of exergaming on physical fitness, body composition and perceived enjoyment in elementary school children. Methods: Sixty-four male students (mean age 9.5 years) were randomly assigned to an exergaming group (EG, n = 32), engaging in Kinect Adventures three times a week for six months, or a control group (CG, n = 32), which continued standard physical education. Assessments were conducted pre- and post-intervention, including anthropometric measures, physical fitness tests (standing long jump, countermovement jump, sit-and-reach, 20-m sprint), aerobic fitness (20-m shuttle run), and perceived enjoyment measured with the PACES scale. Results: The EG demonstrated significant reductions in body weight, BMI, and relative fat mass compared to the CG (*p* < 0.01). Improvements in physical fitness were evident in EG, with increases in standing long jump distance (+12.8%; *p* < 0.0001), countermovement jump height (+65%, *p* < 0.0001), and flexibility (+75%; *p* < 0.0001). Aerobic fitness improved significantly in EG (+87.8m) compared to CG. Perceived enjoyment was notably higher in EG, especially at week three, compared to CG (69.3 ± 5.8 vs. 44.2 ± 11.6; *p* < 0.0001). Conclusions: Exergaming offers benefits for physical fitness and body composition in children, while also enhancing enjoyment. Incorporating exergames into physical education programs could be an effective strategy for addressing childhood obesity, improving physical skills, and increasing student enjoyment, encouraging long-term physical activity adherence.

## 1. Introduction

Several systematic reviews have invariably highlighted the significant benefits of regular physical activity (PA) in improving body composition, physical fitness, and cardiovascular risk profiles among children and adolescents [1,2,3,4]. Regular physical activity is indispensable for healthy growth and development, strengthening muscles and bones, improving cardiovascular health, and more favorable body composition. It also plays a significant role in mental health, reducing symptoms of anxiety and depression, and improving self-esteem and cognitive function [5].

Despite the well-documented benefits of PA, physical inactivity remains a major public health concern among children and adolescents [6]. Various factors contribute to this worrying trend, including increased screen time from televisions, computers, and mobile devices, which encourages sedentary behaviors [7]. Additionally, many children and adolescents lack of access to safe and adequate play environments, such as parks, playgrounds, and recreational facilities, which further limits their opportunities for engaging in PA [3]. Moreover, societal changes, such as urbanization and increased academic pressures, have also played significant roles in reducing the overall PA levels among young people, exacerbating this critical public health issue [8,9]. Consequently, there is an urgent need for preventive and sustainable interventions to promote PA among young people [10]. These multifaceted interventions should involve schools, communities, and families to create supportive environments that encourage active lifestyles [11].

An appropriate environment and perceived enjoyment during PA are crucial factors influencing participation and sustained engagement in childhood [12]. Enjoyment is the most frequently cited intrinsic motivator for children to participate in PA [13,14]. Research also suggests that PA is seen as more enjoyable when children are encouraged to try a range of new activities or different variations of familiar exercises [15,16]. An innovative and motivating approach to promote PA and increase enjoyment, especially in children and adolescents reluctant to engage in PA, can be exergames [17,18]. Exergames combine physical exercise with entertaining video gameplay [19,20] and are therefore considered an important bridge between players’ enjoyment and PA promotion [17,21,22,23]. These games can offer a fun and engaging way to meet the PA requirements while also catering to children’s interests in technology and gaming.

Incorporating exergames into school PA programs or as part of home activities can provide the necessary motivation for children to be more physically active. By making exercise enjoyable and interactive, exergames have the potential to improve children’s physical fitness, motor skills, and overall health, while also fostering a positive attitude towards lifelong PA [23,24]. Recent studies have consistently shown that playing exergames increases energy expenditure more than sedentary behaviors [25,26,27]. Additionally, energy expenditure during exergaming may surpass that of other forms of PA [28,29,30]. From a psychological perspective, exergames have proven to be more enjoyable than traditional exercises, watching television, or playing regular video games, particularly in overweight children [23,31,32]. Research also suggests that exergaming can positively influence children’s moods [33]. Given their popularity among youth, researchers and educators advocate for exergames as tools to engage students in their digital culture and promote a healthy, active lifestyle, especially for those who may be less inclined toward conventional PA [12,18,34]. A study by Lwin and Malik [35] demonstrated that integrating exergaming with health messages in physical education classes has the potential to enhance attitudes and behaviors related to PA, particularly among elementary school children. However, the extent to which exergames can enhance children’s musculoskeletal fitness and increase overall activity levels remains uncertain. Moreover, existing literature highlights significant differences among exergames currently available on the market [18]. Most exergames typically elicit only mild to moderate physical activity and often fail to achieve the intensity levels necessary for significant physiological adaptations [36,37].

Therefore, this study aimed to assess the impact of an intervention utilizing exergames on fundamental motor skills such as strength, endurance, speed, and flexibility in children. By comparing these skills with those of their peers from the same school, we were able to accurately determine the effects of the exergames program.

## 2. Materials and Methods

### 2.1. Participants

Sixty-four male students, aged between 9 and 10 years, with an average age of 9.5 ± 0.7, from an elementary school in Italy, participated in this study. A priori power analysis (G*power, Heinrich Heine University, Düsseldorf, Germany) determined that a minimum of 45 participants would provide sufficient power (0.8) to detect differences. A large effect size of 0.4 (Cohen’s f) and an alpha level of 0.05 were assumed. Therefore, the number of participants in the present study exceeds 40%.

The inclusion criteria required that participants (a) have no disabilities or musculoskeletal, cardiovascular, neurological, respiratory diseases or dysfunctions, cognitive, or mental disabilities, and (b) have no commitments that would prevent them from fully participating in the study or completing the required assessments. The exclusion criteria for the participants included (a) any previous experience using Kinect Adventures^®^ or other similar exergames, to ensure a more homogenous sample; and (b) any significant behavioral issues that could disrupt the study.

Teachers and parents were provided with comprehensive information about the study and written parental or legal representative informed consent was obtained before any student was included. The study was conducted by the Helsinki Declaration (document 111/3976/88, July 1990). The University’s Research Ethics Committee approved the study (N.1/2021in 7 January 2021). Children and their parents received an informative letter about the study, including an informed consent form.

A randomization procedure of the classrooms (a computer-generated list of random numbers using SPSS, version 24) was carried out by the two lead researchers (A.M. and S.M., co-authors).Participants were randomly assigned to one of two groups: an exergames group (EG; n = 32), which followed a structured PA program based on exergames, and a control group (CG group; n = 32), which continued their regular physical education classes without any change in PA habits. Children who were not part of the study continued with their regular physical education classes as scheduled by the school or engaged in exergames as pedagogical content after the study concluded. Before the study began, neither group engaged in regular PA outside of the mandatory physical education classes at school.

A total of 64 participants completed the entire 6-month intervention period, with no attrition during the study.

### 2.2. Experimental Design

The study aimed to evaluate the effects of regular exergaming on the physical composition and the physical and athletic abilities of children. Over a six-month intervention period, participating students engaged in exergaming sessions three times a week for 45 min each. These sessions took place outside of school hours, ensuring they did not replace the children’s mandatory physical education classes. The intervention classes were held as separate, structured sessions designed specifically for the study and were conducted, then, in addition to the participants’ regular physical education lessons. These sessions (Kinect Adventures**^®^** exergames, Xbox Game Studios, Redmond, WA, USA) were complementary, ensuring that participants in the experimental group received additional physical activity beyond their usual school curriculum; in contrast, the control group only participated in the standard physical education lessons as part of their school curriculum, without additional intervention sessions.

Before the intervention (T0) and after a six-month intervention period (T1) we assessed physical and athletic performance to evaluate the effectiveness of the proposed activities. Results were compared with those of children of the same age who did not participate in additional intervention sessions, but attended compulsory physical education classes at school. Anthropometric measurements, including weight and height, were also assessed to calculate the body mass index (BMI). Throughout the intervention period, perceived enjoyment was assessed twice (week three and week twenty) using the “Physical Activity Enjoyment Scale” (PACES), at the end of the exergaming session or PE class.

### 2.3. The Exergame Procedure

Regarding the selection and application of games for the children to play, the choice fell on Kinect Adventures released by Xbox Game Studios. This game offers, in addition to a free play option (which allows players to freely tackle any of the five activities included on the disc), a sort of “story mode”: an organized progression that takes the player (or players) through a series of “extravagant” adventures. This choice was motivated by its popularity and proven effectiveness in engaging participants in fun and engaging PA.

The participants engaged in Kinect Adventures three times per week for six months. Each session lasted approximately 45 min, with the intensity of the games varying from moderate to high.

The games involved movements combining resistance and aerobic activity, with a focus on dynamic exercises improving coordination, balance, and muscular strength.

The activities within Kinect Adventures**^®^** include a variety of physical challenges designed to promote movement and coordination. For example, players may engage in river rafting, where they navigate rapids by physically leaning and paddling, thereby enhancing their balance and upper body strength. Other activities, such as space jumping and obstacle courses, require players to jump, duck, and maneuver around obstacles, which not only improves cardiovascular fitness but also encourages agility and reaction time. The incorporation of these varied activities provides a comprehensive approach to physical fitness, engaging different muscle groups and promoting a holistic development of children’s physical abilities.

Caloric expenditure was estimated using a wearable heart rate monitor (Polar Electro Oy, Kempele, Finland). During active gameplay with Kinect Adventures**^®^**, children burn between 4 to 8 calories per minute, depending on the intensity of the game and the level of engagement. For a 30-min session, this translates to approximately 120 to 240 calories burned, based on body weight, and overall physical activity levels of the participants.

It was recommended that the activity be practiced in the afternoon, immediately after school hours, to ensure that the children were adequately rested and ready to actively participate in physical exercise. The exercise sessions were led by the same group of physical education teachers who are familiar with the students. This consistency in instruction ensures that the children receive a cohesive and supportive experience during both their regular physical education classes and the additional exergame sessions.

### 2.4. Measures

The study protocol included an initial assessment at the beginning of the study (T0) and a final assessment after 6 months of intervention (T1). Data collection took place in the gym at nine a.m., with measurements conducted by the same “blinded” evaluators.

#### 2.4.1. Anthropometrics

The children’s height was measured using a Seca stadiometer (Seca, Hamburg, Germany) with an accuracy of 0.1 cm, and their weight was measured using an Omron scale (OMRON Healthcare, Inc., Kyoto, Japan) with an accuracy of 0.1 kg. Both body weight and height were measured twice using standard methods. Waist circumference (WC) was also measured twice at the iliac crest using a non-elastic tape measure. BMI is obtained by dividing the weight (in kilograms) by height (in meters squared) [38]. The relative fat mass (RFM, pediatric) was calculated according to the formula (22 × height (m)/WC (m) + (5 × sex) using sex = 0 for boy and 1 for girl) [39]. The Woolcott and Bergman Relative Fat Mass (RFM) formula [39] was validated using data from the U.S. National Health and Nutrition Examination Survey (NHANES), specifically from participants aged 8 to 19 years. The study population included 10,390 children and adolescents of various ethnic backgrounds, ensuring a broad representation across different groups. The formulas were evaluated against Dual Energy X-ray Absorptiometry (DXA) measurements, a gold standard for estimating body fat. RFM was validated for children aged 8 to 14, showing better accuracy than BMI in estimating whole-body fat percentage and diagnosing obesity, particularly among boys. 

The cut-off points of RFM are more accurate than BMI to provide an estimation of whole-body fat percentage among children.

#### 2.4.2. Physical Fitness

Before the intervention, we selected specific tests to evaluate the effectiveness of PA programs and motor skills of children and adolescents aged 5 to 17 [40]; it covered various aspects of physical fitness and included standing long jump test, countermovement jump test, sprint tests and sit-and-reach test [41,42].

Each test was specifically adapted to suit children’s motor skills and physical size.

The tests were consistently adapted for 9-year-old children by simplifying the instructions, offering practice tests, and including appropriate rest periods suited to their physical development and endurance. These changes ensured that the tests were suitable for accurately assessing their physical fitness.

The scores from these tests evaluate improvements following the intervention and provide comparisons with the reference population. The tests to evaluate physical-athletic performance were conducted in a gym to ensure a controlled and suitable environment for accurate assessment and implementation of the physical activity program.

The standing long jump test is a valid, robust, and practicable test to assessing lower limb explosive strength and physical [43]. The performance followed a standard operating procedure [44]. The best score of the three attempts was retained for investigation.

The countermovement jump test is a widely used valid and reliable method to measure leg power and explosiveness coordination abilities [45], essential components of musculoskeletal fitness and motor skill development in children [46]. Children were asked to step down from a standing position with their hands on their hips and then jump as high as possible during the next phase. Jump height was calculated using a jump photocell system (Microgate, Bolzano, Italy), a validated tool for jump height assessment [47]. After a familiarization test, the best result of two jump trials was used for analysis.

The sit-and-reach test is one of the most common field tests in physical fitness batteries to assess the flexibility of subjects [44]. Following the guidelines, children sat, with their knees extended and their feet resting on vertical supports. They were asked to reach their hands forward as far as possible. The best score of the two attempts was recorded.

Sprint tests are commonly used in schools to determine power and strength [48,49]. Twenty-meter sprint times were assessed using portable electronic timing gates. Children were asked to perform two 20-m sprints separated by three minutes of rest. All students started with their front foot positioned 0.5 m behind the starting line and were instructed to perform both sprints with maximal effort. The best result of the two sprints was used for analysis.

#### 2.4.3. Aerobic Fitness

Aerobic fitness was assessed using a 20 m shuttle run test, as a reliable method to predict aerobic fitness in children [50,51]. Children were asked to run back and forth between two lines 20 m apart. An audio signal emitted specific frequencies (Microgate Srl, Bolzano, Italia) and a co-author was used to set the pace. Children were asked to complete as many shuttles as possible, initially at a speed of 8.5 km/h and increased by 0.5 km/h every minute. When a child stopped due to fatigue or after two consecutive failures to complete a shuttle in the allotted time, the test was stopped. The number of shuttles and phases completed were recorded for each student to predict aerobic fitness.

#### 2.4.4. Heart Rate

Heart rate (HR) was continuously monitored during the exergaming sessions using a Polar HR monitor (Polar Electro Oy, Kempele, Finland). The average HR (HRmean) for each student was calculated by averaging their HR readings across all sessions. To estimate the percentage of maximal HR achieved, individual maximal HR (HRmax) was calculated using the formula HRmax = 208 − 0.7 × age [52]. Throughout the exergaming sessions, students achieved a mean HR of 175.3 ± 2.1 bpm, which corresponded to 87.4 ± 0.2% of their estimated HRmax.

### 2.5. Enjoyment

The PACES questionnaire consisting of 16 bipolar statements was used to measure perceived pleasure during the PE lesson or during the game [53]. On a five-point scale, students rated how they felt about the exercise they had just performed. An average score was calculated for each session. Before answering the questionnaire, students were asked to answer as truthfully as possible. The PACES is widely used and appropriate for children [18,54]. Furthermore, it has been validated as a reliable and valid measurement instrument, with a good internal consistency between 0.92 and 0.93 [53].

### 2.6. Statistical Analyses

All results here presented are means ± standard deviations. Statistical analyses were performed using SPSS (version 24.0, IBM; Chicago, IL, USA). Differences in subject characteristics between groups were assessed using an independent samples *t*-test. Homogeneity of variances was tested using Levene’s test, which indicated that the assumption of homogeneity of variances was met (*p* > 0.05), which allowed us to proceed with parametric statistical methods such as ANOVA. To evaluate changes over time within each group (pre- vs. post-treatment, T0 vs. T1), paired samples *t*-tests were performed on the data from the exergaming and control groups. To further identify significant differences in study outcomes between the two groups (exergaming vs. control) over time, a series of two-way ANOVAs with repeated measures (time: T0 vs. T1) were conducted. Post-hoc analyses with Tukey HSD correction were also performed, and effect sizes were measured using partial eta squared (η²). Effect sizes were categorized as small, medium, and large with values of 0.01 ≤ 0.06, 0.06 < 0.14, and ≥0.14, respectively. The significance level was set at *p* < 0.05.

## 3. Results

### 3.1. Effect of Exergames on Body Composition

There were no significant differences between the EG and CG in terms of average weight, height, BMI, and relative fat mass (RFM) at T0 (*p* > 0.05, according to Student’s *t*-test; see Table 1).

However, children participating in the structured physical activity program based on exergames reduced body weight and BMI compared to the control group (*p* < 0.01 according to Student’s *t*-test, Table 1).

### 3.2. Physical Fitness

**Figure 1:**
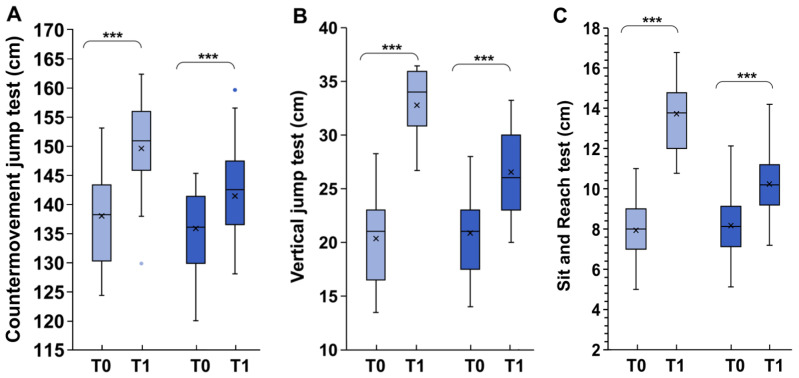
Differences in countermovement jump test (**A**) vertical jump test (**B**) and sit-and-reach test (**C**) between exergames (light blue) and control groups (dark blue). T0 represents the start of the experiment; T1 represents the end of the experiment (six months later). In this representation, the central box covers the middle 50% of the data values, between the upper and lower quartiles. The bars extend to the extremes, the central line represents the median, and the cross indicates the mean value. The individual points represent those values that are beyond 1.5 times the interquartile range beyond the central box. *** *p* < 0.0001 values were obtained using a paired *t*-test comparing values from the same subjects before and after the experiment.

We used the standing long jump test to assess leg power, muscle strength, and motor coordination [43,55]. Before the observation period (T0), there were no significant differences between CG and EG (150.20 ± 6.1 cm and 149.92 ± 6.8 cm, for EG and CG, respectively, *p* > 0.05). In T1, the increase in jump distance was significantly greater in EG; in this group, the jump distance increased by 12.8% by the end of the study period. In contrast, the CG showed a 4.1% increase only (Figure 1A). There was an overall significant ‘Time x Group’ difference between EG and CG (*p* < 0.01; F = 7.03; η^2^ = 0.038).

Using the countermovement jump test, we assessed the difference in strength and coordination capabilities between the two groups of children [45]. In T0, vertical jump measurement for EG was 20.35 ± 5.72 cm, and 20.71 ±6.26 cm for CG, therefore showing no significant difference between the groups (*p* > 0.05). Although both groups exhibited significant increases in jump, it was significantly higher in the EG (+65% for EG and +30% for CG); thus, in T1, there was an overall significant difference between the EG and the CG (*p* < 0.0001; F =28.21; η^2^ = 0.364) (Figure 1B).

To quantify flexibility, the sit-and-reach test was selected because it is frequently used among children and adolescents [45,56].

Again, there were no differences between EG and CG in sit-and-reach performance before (*p* > 0.05; 7.94 ± 1.4 cm and 7.97 ± 1.5 cm, CG and EG, respectively). At the end of the study period, significantly better changes in performance in the treated group were recorded (*p* < 0.001; F = 103.553; η^2^ = 0.715); in fact, there was an increase in flexibility of 75% (from 7.97 ± 1.5 cm to 13.97 ± 1.68 cm, *p* < 0.001, Tukey HSD) in the EG and of only 28% (from 7.94 ± 1.4 cm to 10.08 ± 1.53 cm, *p* < 0.001 Tukey HSD) in the control group.

**Figure 2:**
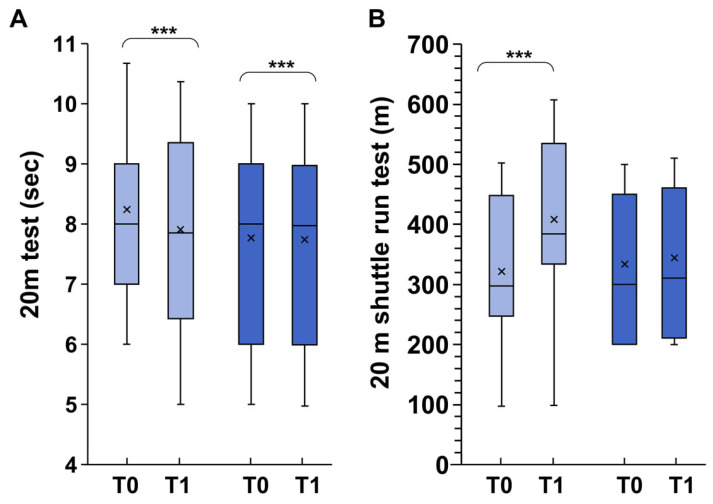
Differences in 20 m test (**A**) and 20 m shuttle run test (**B**) between exergames (light blue) and control groups (dark blue). T0 represents the start of the experiment; T1 represents the end of the experiment (six months later). In this representation, the central box covers the middle 50% of the data values, between the upper and lower quartiles. The bars extend to the extremes, the central line represents the median, and the cross indicates the mean value. *** *p* < 0.001 values were obtained using a paired *t*-test comparing values from the same subjects before and after the experiment.

The 20 m test is an excellent tool for assessing speed and anaerobic power in 9-year-olds [57,58]. At T0, there was no significant difference in the mean running time between EG (7.68 ± 1.8 s) and CG (7.88 s ± 2.2 s; *p* > 0.05) (Figure 2A). In EG at T1 there was an overall significant decrease in time (*p* < 0.0001). Post hoc test revealed no significant group–time interactions (*p* = 0.560; F = 0.690; η^2^ = 0.014).

### 3.3. Aerobic Fitness

The level of aerobic fitness was assessed using the multistage 20 m shuttle run test. Significant group–time interactions (*p* = 0.002, F = 5.393; η^2^ = 0.099) were observed for the shuttle run test, with a significant increase in distance covered in the EG (+ 80.51 ± 10.66 m, *p* = 0.004). The CG showed no significant changes (an increase of 10.4 ± 2.59 m; *p* = 0.485).

### 3.4. Perceived Enjoyment

**Figure 3:**
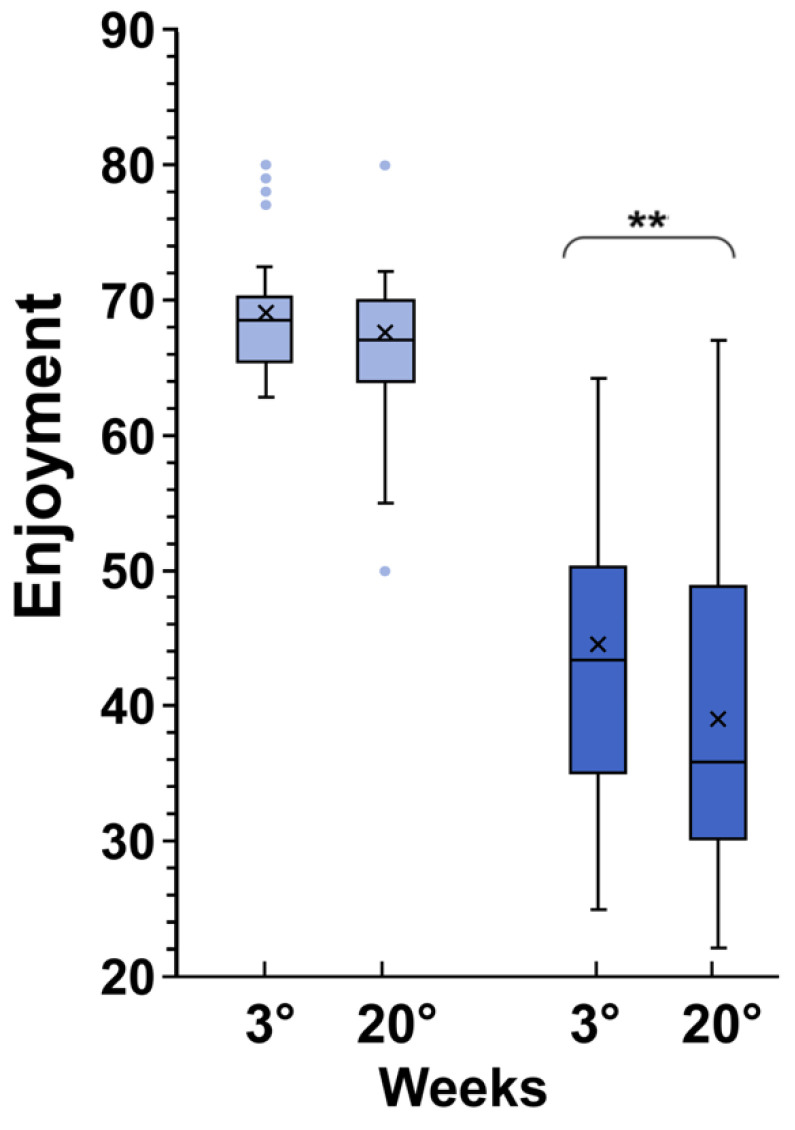
Differences in perceived enjoyment during an exergaming session in weeks three and twenty in exergames (light blue) and control groups (dark blue). In this representation, the central box covers the middle 50% of the data values, between the upper and lower quartiles. The bars extend to the extremes, the central line represents the median, and the cross indicates the mean value. The individual points represent those values that are beyond 1.5 times the interquartile range beyond the central box. ** *p* < 0.0001 values were obtained using a paired *t*-test comparing values from the same subjects before and after the experiment.

The mean PACES score in EG in week three was significantly higher than in CG at the same week (69.3 ± 5.8 versus 44.2 ± 11.6; *p* < 0.0001 by unpaired *t*-test). After 20 weeks in the EG group, no significant differences in perceived enjoyment were observed (*p* = 0.101); on the contrary, in the CG, the mean PACES score decreased significantly (*p* < 0.0061, by paired *t*-test). (Figure 3).

## 4. Discussion

The study assessed the impact of exergames on motor skills and perceived enjoyment in elementary school children. The study found significant improvements in body weight, body mass index (BMI), leg power, muscle strength, flexibility, and aerobic fitness in children involved in the exergame-based structured physical activity program compared to the control groups. In addition, the exergame program also led to better-perceived enjoyment scores compared to classic physical education classes.

Childhood obesity, a growing concern worldwide, has reached alarming levels in many countries, posing serious challenges to public health [59]. The prevalence of obesity among children has more than tripled in recent decades, leading to increased risks of chronic diseases such as type 2 diabetes, hypertension, and cardiovascular disease at younger ages [60]. This epidemic is driven by various factors, including unhealthy dietary habits, sedentary lifestyles, and environmental influences that promote calorie-dense, nutrient-poor food choices [3,61]. Engaging children in regular physical activities helps increase energy expenditure, which is essential for maintaining a healthy weight and preventing excessive fat accumulation [1,19,62]. Studies have shown that school-based interventions that incorporate regular physical activity can significantly reduce the prevalence of obesity in children by improving their overall fitness levels and reducing their BMI [11,38,63,64,65].

Here, interestingly, children involved in the exergame-based structured physical activity program showed a decrease in both total body weight and BMI compared to the control group.

In fact, exergames combine physical activity with engaging, game-like experiences, encouraging children to be more active and burn calories [66]. Previous studies have shown that these interactive games can significantly increase energy expenditure in children, making them an enjoyable and effective tool for weight management [30]. Additionally, maintaining a healthy weight during childhood is essential for preventing obesity-related health problems in adulthood, such as type 2 diabetes and cardiovascular disease [67]. Early intervention through enjoyable physical activities such as exergaming can help establish lifelong healthy habits, reducing the risk of chronic diseases later in life [17]. Unfortunately, children often discontinue physical activity programs because they become bored and experience no sense of enjoyment, which decreases their motivation to continue [57,58]. The lack of enjoyment during exercise can lead to lower adherence and increased dropout rates in these programs [45,56]. Since studies suggest a correlation between enjoyment and increased engagement in physical activity [68], enjoyable exergames may positively influence future physical activity behaviors. Furthermore, enjoyment has been recognized as a key factor in maintaining engagement in physical activity among children and adolescents [69,70,71]. From our results, we can assume that Kinect Adventures represents a tool to implement joy and keep most students engaged for a long time. The results are relevant since a decrease in physical activity can be observed especially in pre-pubertal age and early puberty, which is attributed to a lack of perceived enjoyment of physical activity [72,73]. Our findings align with previous studies that have demonstrated high levels of perceived enjoyment during classroom and out-of-school exergaming interventions [18,73,74,75].

Therefore, exergames that integrate technology with physical activity have shown promise in engaging children in exercise. These games align with children’s interests, making exercise more engaging and increasing the likelihood of sustained participation [26,34,49]. Thus, the interactive and competitive features of exergames motivate children to be active, helping them overcome the challenges associated with traditional forms of exercise [17].

From our experience, Kinect Adventures, for its fast-paced nature, is widely regarded as one of the most enjoyable and engaging exergames available on the Xbox Kinect. This game leverages motion-sensing technology to offer an interactive experience where players can physically jump, dodge, and lean to navigate various virtual adventures. Whether rafting down wild rivers, navigating obstacle courses, or popping bubbles in space, the game provides a fun and immersive way to stay active.

In addition, exergames have been shown to improve various health outcomes, such as cardiovascular fitness, muscle strength, and coordination [20].

One of the key advantages of exergames is their ability to provide a customizable and adaptable form of exercise that can be tailored to suit individual preferences and fitness levels. This flexibility makes them particularly effective in sustaining long-term engagement in physical activity among children, who may otherwise lose interest in traditional forms of exercise [76].

In our study, children who also participated in exergaming showed greater increases in jump distance, flexibility, and shuttle run performance after six months, while those who continued to attend PE classes only showed minimal changes. Both groups improved in countermovement jumping, but EG had a significantly greater increase. The significant improvements observed in the physical fitness indicators of the exergame group can be attributed to the diverse and dynamic nature of the activities involved in the exergames. The combination of aerobic exercises (such as running, jumping, and dodging obstacles), coordination tasks, and strength-based movements (like squats and arm movements) provided a comprehensive workout targeting multiple fitness components. These activities engage both the cardiovascular system and musculoskeletal systems, promoting endurance, strength, and flexibility. The interactive and enjoyable format of the games likely encouraged sustained effort and participation, which is critical for eliciting meaningful physiological adaptations in children. The improvement in shuttle run performance, for example, suggests enhanced aerobic capacity, while gains in jumping ability reflect increases in leg strength and motor coordination. Furthermore, the competitive and immersive nature of exergames may have heightened motivation and effort, resulting in greater engagement and, consequently, more significant fitness improvements compared to traditional physical education.

Improving several health outcomes such as cardiovascular fitness, muscle strength, and coordination is essential for 9-year-old children, as these elements are critical for their overall physical development and well-being. Cardiovascular fitness supports endurance and the ability to engage in prolonged physical activities; this is vital for maintaining a healthy weight and promoting heart health [77,78]. Increasing muscle strength not only improves physical performance and reduces the risk of injury but also supports the development of motor skills and physical confidence [79,80].

Addressing these aspects of health help establish a foundation for an active lifestyle and promotes overall well-being, setting the basis for healthier habits in adulthood [81].

In conclusion, exergames represent a promising tool for promoting physical activity among children by leveraging the engaging and enjoyable aspects of gaming. By aligning exercise with children’s interests and making it a fun experience, exergames have the potential to foster healthier lifestyles and combat the growing epidemic of childhood obesity and sedentary behavior [30]. Additionally, exergames can be played indoors, making them a convenient option for physical activity during adverse weather conditions or in environments where outdoor play space is limited.

Of course, our study is not without limitations. While the study’s sample size exceeds the minimum requirement determined by power analysis, it is relatively small and limited to a single school in Italy. This may affect the generalizability of the findings to other populations, schools, or geographic locations. Because the study included only male participants, sex differences in physical fitness and response to exergaming were not explored.

In addition, a longer-term intervention or other types of exergames could offer different levels of engagement and physical challenge, which may affect overall outcomes.

Finally, physical fitness assessments were conducted in a controlled environment (gym) that may not fully replicate real-world conditions; results may vary in more varied or less controlled settings.

Addressing these limitations in future research could provide a clearer understanding of the impact of exergaming on physical fitness and enjoyment in children.

## 5. Conclusions

The results indicate that exergaming is enjoyable and can evoke some benefits for physical fitness and physical activity. Our study encourages the introduction of exergaming systems in primary and secondary schools to improve conditional skills and increase student enjoyment. Finally, exergames can be excellent allies for an active lifestyle, but they should not completely replace traditional physical activity.

## Figures and Tables

**Table 1:** Physical Characteristic of children.

**Exergames group (EG, n = 32)**	**T0**	**T1**
Age, y	9.5 ± 0.6	9.6 ± 0.7
Weight, Kg	33.6 ±2.5	31.3 ± 1.7 *
Height (cm)	138.5 ± 3.8	139.1± 3.9
BMI	17.9 ± 3.1	16.3± 0.9 *
RFM	26.2 ± 2.7	24.5 ± 1.6 *
**Control group (CG, n = 32)**	**T0**	**T1**
Age, y	9.5 ± 0.8	9.7 ± 0.8
Weight, Kg	32.9 ± 3.3	33.9 ± 2.4 *
Height (cm)	137.9 ± 3.8	139.1± 4.3
BMI	17.1 ± 3.3	17.7. ± 5.6 *
RFM	25.8 ± 1.6	26.3 ± 1.6

Abbreviation: BMI, body mass index (calculated as weight in kilograms divided by height in meters squared). RFM, relative fat mass. * *p* < 0.01 between T0 and T1 by paired *t*-test.

## Data Availability

The raw data supporting the conclusions of this article will be made available by the authors upon request.
